# Body Configuration as a Predictor of Mortality: Comparison of Five Anthropometric Measures in a 12 Year Follow-Up of the Norwegian HUNT 2 Study

**DOI:** 10.1371/journal.pone.0026621

**Published:** 2011-10-20

**Authors:** Halfdan Petursson, Johann A. Sigurdsson, Calle Bengtsson, Tom I. L. Nilsen, Linn Getz

**Affiliations:** 1 Research Unit of General Practice, Department of Public Health and General Practice, Norwegian University of Science and Technology (NTNU), Trondheim, Norway; 2 Department of Family Medicine, University of Iceland, and Centre of Development, Primary Health Care of the Capital Area, Reykjavik, Iceland; 3 Department of Primary Health Care, The Sahlgrenska Academy, University of Gothenburg, Gothenburg, Sweden; 4 Department of Human Movement Science, Norwegian University of Science and Technology (NTNU), Trondheim, Norway; Innsbruck Medical University, Austria

## Abstract

**Background:**

Distribution of body fat is more important than the amount of fat as a prognostic factor for life expectancy. Despite that, body mass index (BMI) still holds its status as the most used indicator of obesity in clinical work.

**Methods:**

We assessed the association of five different anthropometric measures with mortality in general and cardiovascular disease (CVD) mortality in particular using Cox proportional hazards models. Predictive properties were compared by computing integrated discrimination improvement and net reclassification improvement for two different prediction models. The measures studied were BMI, waist circumference, hip circumference, waist-to-hip ratio (WHR), and waist-to-height ratio (WHtR). The study population was a prospective cohort of 62,223 Norwegians, age 20–79, followed up for mortality from 1995–1997 to the end of 2008 (mean follow-up 12.0 years) in the Nord-Trøndelag Health Study (HUNT 2).

**Results:**

After adjusting for age, smoking and physical activity WHR and WHtR were found to be the strongest predictors of death. Hazard ratios (HRs) for CVD mortality per increase in WHR of one standard deviation were 1.23 for men and 1.27 for women. For WHtR, these HRs were 1.24 for men and 1.23 for women. WHR offered the greatest integrated discrimination improvement to the prediction models studied, followed by WHtR and waist circumference. Hip circumference was in strong inverse association with mortality when adjusting for waist circumference. In all analyses, BMI had weaker association with mortality than three of the other four measures studied.

**Conclusions:**

Our study adds further knowledge to the evidence that BMI is not the most appropriate measure of obesity in everyday clinical practice. WHR can reliably be measured and is as easy to calculate as BMI and is currently better documented than WHtR. It appears reasonable to recommend WHR as the primary measure of body composition and obesity.

## Introduction

It is well documented that distribution of body fat is more important than the amount of fat as a prognostic factor for metabolic disturbance, cardiovascular diseases (CVD) and life expectancy [1]–[6]. Central or abdominal fat has been associated with the highest risk [7], with visceral fat being of special importance [8], [9]. Increased waist-to-hip ratio (WHR) as a risk factor for CVD and mortality was first reported from the Swedish population studies in Gothenburg in 1984 where WHR was shown to be a stronger prognostic factor than body mass index (BMI) [3], [4]. These results have repeatedly been confirmed since [10]–[14]. The specific protective effect of increased peripheral (lower body) fat in the form of hip and thigh [15] circumference in contrast to waist girth has also been reported [9], both for men [13], [16] and women [17], [18]. The more recent measure waist-to-height ratio (waist circumference divided by height [WHtR]) disregards the peripheral fat but takes the height into account. Some researchers have found WHtR to be an even stronger predictor of death, CVD [19]–[22] and CVD risk factors [23], [24] than the above mentioned measures. Others have suggested the difference of these anthropometric measures to be insignificant or none at all, regarding predictive ability for CVD [25], [26].

In recent years an increasing amount of knowledge has been gathered regarding the metabolic basis for the special importance of central fat distribution. Various metabolic, endocrine and neural factors appear to influence where the body fat accumulates and how this affects the individual's physiology and disease risk [5], [8], [9], [27].

In the wake of the increasing prevalence of obesity in most parts of the world [28]–[32], there has been a debate about which anthropometric measures are best suited to define and monitor obesity for medical purposes. Despite the evidence of abdominal obesity being far more important than body weight regarding mortality and CVD, definitions of overweight and obesity based on BMI continue to be the most widely used measures in publications, including clinical guidelines designed for individual, preventive or therapeutic counselling [28], [33]–[35]. Many guidelines mention WHR and/or waist circumference as interchangeable with BMI although the correlation is questionable [10], [11], [21], [23]. This practical approximation decreases the specificity of the obesity definition and undermines the predictive precision of CVD risk estimates.

The aim of this study was to further clarify the associations of anthropometric indicators of obesity and body composition (BMI, WHR, WHtR, waist circumference, and hip circumference) with overall mortality, and specifically with CVD mortality.

## Methods

### Study population

All adults aged 20 years or older and living in Nord-Trøndelag County in Norway were in 1995 to 1997 invited to participate at the second wave of the Nord-Trøndelag Health Study (HUNT 2). Overall, 74% of women (34,786) and 65% of men (30,575) chose to participate. The HUNT 2 population is ethnically homogenous (dominated by individuals of Nordic origin) and has been considered representative of the total Norwegian population regarding demography, socio-economic factors, morbidity and mortality, including mortality from CVD [36]. The HUNT 2 study has been described in detail elsewhere [36] (see www.ntnu.no/hunt/english).

For the purpose of the present analysis, 3,138 participants aged 80 years or more at baseline (1,231 men and 1,907 women) were excluded. Individuals with established CVD at baseline (self-reported myocardial infarction, stroke or angina pectoris) were excluded, 4,571 in total (2,780 men and 1,791 women), as well as 681 person with missing data on one or more of the following variables: height, weight, waist circumference, and hip circumference. Our calculations are thus based on information from 56,971 individuals (26,461 men and 30,510 women) aged 20–79 years who were without any known CVD at baseline. Baseline characteristics are depicted in the supporting information (Table S1)

### Study variables

In the HUNT 2 study, height and weight were measured with participants wearing light clothes without shoes; height to the nearest 1.0 cm and weight to the nearest 0.5 kg. Based on these measures we calculated BMI as weight in kg divided by the squared value of height in meters. Waist and hip circumferences were measured with a steel band to the nearest 1.0 cm with the participant standing and with the arms hanging relaxed. The waist circumference was measured horizontally at the height of the umbilicus, and the hip circumference was measured likewise at the thickest part of the hip [36]. When analysing the anthropometric measures, we aimed at using clinically recognisable categorisations, rather than percentiles. BMI was categorised according to WHO definitions [28], the waist circumference categories were defined with 10 cm interval, and the hip circumference categories with 5 cm interval. The WHR and WHtR were, however, categorised by quintiles.

In the present analysis smoking was defined as daily smoking of cigarettes, cigars or a pipe. Smoking status was defined as unknown, current smoker, former, or never smoker. Levels of recreational physical activity were defined as self-reported number of hours spent on hard or light activity during one week: no activity; <3 h light activity; ≥3 h light activity or <1 h hard activity; ≥1 h hard activity; unknown.

### Follow-up

The personal identity number of Norwegian citizens enabled linkage of HUNT 2 participant data to the Cause of Death Registry at Statistics Norway (information on www.ssb.no/english/). For the present analysis, each participant contributed person-time from the date of clinical examination (August 1995–June 1997) until the date of death or end of follow-up (December 31^st^ 2008). The mean follow-up time was 12.0 years, in total 684,644 person-years. Death from CVD was defined by the International Classification of Disease code for the primary diagnosis of death (ICD-9: 390–459; ICD-10: I 00-I 99).

### Ethics statement

Each participant in the HUNT study signed a written consent regarding the screening and the use of data for research purposes as well as to linking their data to other registers (subject to approval of the Norwegian Data Inspectorate). The study was approved by the Norwegian Data Inspectorate and by the Regional Committee for Ethics in Medical Research.

### Statistical analysis

We used Cox proportional hazard models to compute hazard ratios (HRs) for overall mortality and CVD mortality associated with different levels of each anthropometric measure. Precision of the estimated associations was assessed by a 95% confidence interval. Departure from the proportional hazards assumption was evaluated by Schoenfeld residuals and log-minus-log plots. An interaction term between time and the appropriate variables was added to the model if the proportional hazards assumption did not hold.

We analysed the HR for participants with BMI below 18.5 kg/m^2^ (104 men and 314 women) for comparison with the other BMI categories but excluded them from further analysis due to the potential of reverse causality (a J-shaped mortality curve) [37], [38].

We calculated sex specific standard deviation (SD) scores for each of the anthropometric variables and estimated the HR associated with an increase of one SD.

We analysed the data separately for men and women, and all associations were adjusted for potential confounding effects of age, smoking status and recreational physical activity. We conducted sensitivity analyses involving three additional models (Model 2–4). Model 2 included the same covariates as the main model but excluded participants with unknown smoking status. Model 3 was adjusted for age, smoking, and physical activity (as our main model) in addition to self-reported diabetes mellitus and weekly alcohol consumption (abstinence, 0–2 glasses [units], 2.1–5 glasses, 5.1–8 glasses, >8 glasses). Model 4 was identical to our main model but excluded the first three years of follow-up to limit the reverse causality effect of undiagnosed diseases.

The “relative informativeness” of each anthropometric measure was evaluated by examining the contributions made to the χ^2^ likelihood ratio statistic in the Cox regression model compared with a model that only contained the confounders, as the χ^2^ statistic can be used as a measure of the improvement of goodness of fit [39]. This was done both in relation with all cause and CVD mortality, respectively.

To further compare the predictive properties of the different anthropometric measures for CVD death, sex-specific net reclassification improvement (NRI) and integrated discrimination improvement (IDI) were computed when adding each anthropometric measure to two different prediction models. Model A included age as the only predictive variable, while Model B included age, smoking status, systolic blood pressure, and total cholesterol. For each model three different NRI calculations were done, using two (<5%, ≥5%), three (<1%, 1–9%, ≥10%), and four (<1%, 1–4%, 5–9%, ≥10%) levels of risk of CVD death, respectively.

In addition, we conducted an analysis of the anthropometric measures stratified by age (above and below 60 years). Finally, mutually adjusted analyses were conducted for waist and hip circumference, as well as for BMI and WHR.

All statistical tests were two-sided and all analyses were performed using Stata for Windows (Version 11 StataCorp LP, TX, USA).

## Results

We present the risk of death from all causes and from CVD among men and women aged 20–79 (Tables 1 and 2) with absolute numbers and HRs in association with each of the anthropometric measures studied, after adjusting for age, smoking and physical activity. For men, WHR and WHtR had the highest (and similar) predictive power, both regarding mortality from CVD and from all causes, followed by waist circumference (Table 1). BMI had considerably weaker association, with HRs only reaching statistical significance for death from CVD but not overall mortality.

**Table 1:** Risk of death from all causes and from cardiovascular disease among men aged 20–79; associations with anthropometric measures.

		All causes			Cardiovascular disease		
Anthropometric measures	No. of persons	No. of deaths	Adjusted^a^ HR (95% CI)	*P*_trend_	No. of deaths	Adjusted^a^ HR (95% CI)	*P*_trend_
**Body mass index (kg/m^2^)**							
<18.5^b^	104	31	2.48 (1.73–3.54)		9	2.23 (1.15–4.33)	
18.5–24.9	9,575	970	1.00 (Reference)		300	1.00 (Reference)	
25.0–29.9	13,138	1,320	0.86 (0.79–0.93)		492	1.04 (0.90–1.21)	
30.0–34.9	3,154	445	1.10 (0.98–1.23)		175	1.42 (1.17–1.71)	
≥35.0	490	70	1.34 (1.05–1.72)	0.16	28	1.78 (1.20–2.64)	<0.001
per 5 kg/m^2^	26,357	2,805	1.04 (0.98–1.10)	0.21	995	1.19 (1.08–1.30)	<0.001
per SD (3.4 kg/m^2^)	26,357	2,805	1.02 (0.99–1.06)	0.21	995	1.12 (1.06–1.20)	<0.001
**Waist circumference (cm)**							
<80	1,882	116	1.17 (0.96–1.42)		33	1.06 (0.74–1.53)	
80–89	9,466	723	1.00 (Reference)		233	1.00 (Reference)	
90–99	10,378	1,134	1.01 (0.92–1.11)		404	1.10 (0.93–1.29)	
100–109	3,625	588	1.11 (0.99–1.24)		226	1.27 (1.05–1.53)	
≥110	1,006	244	1.64 (1.41–1.90)	<0.001	99	1.99 (1.56–2.53)	<0.001
per 10 cm	26,357	2,805	1.11 (1.07–1.16)	<0.001	995	1.21 (1.13–1.29)	<0.001
per SD (9.1 cm)	26,357	2,805	1.10 (1.06–1.14)	<0.001	995	1.19 (1.12–1.26)	<0.001
**Hip circumference (cm)**							
<95	2,360	275	1.18 (1.02–1.36)		80	0.96 (0.75–1.25)	
95–99	6,158	639	1.00 (Reference)		225	1.00 (Reference)	
100–104	9,203	925	0.89 (0.80–0.99)		335	0.92 (0.77–1.09)	
105–109	5,471	546	0.86 (0.76–0.96)		200	0.89 (0.73–1.08)	
≥110	3,165	420	1.17 (1.03–1.33)	0.52	155	1.23 (1.00–1.51)	0.24
per 10 cm	26,357	2,805	1.01 (0.95–1.07)	0.76	995	1.11 (1.00–1.23)	0.05
per SD (6.2 cm)	26,357	2,805	1.01 (0.97–1.05)	0.76	995	1.06 (1.00–1.13)	0.05
**Waist-to-hip ratio**							
<0.85	5,301	254	1.07 (0.90–1.26)		75	1.11 (0.82–1.50)	
0.86–0.87	5,126	328	1.00 (Reference)		97	1.00 (Reference)	
0.88–0.89	5,287	493	1.12 (0.97–1.29)		167	1.25 (0.97–1.60)	
0.90–0.93	5,367	646	1.14 (0.99–1.30)		233	1.33 (1.05–1.69)	
≥0.94	5,276	1,084	1.38 (1.21–1.56)	<0.001	423	1.70 (1.36–2.13)	<0.001
per 0.1 unit	26,357	2,805	1.28 (1.20–1.36)	<0.001	995	1.43 (1.29–1.59)	<0.001
per SD (0.06)	26,357	2,805	1.15 (1.11–1.19)	<0.001	995	1.23 (1.16–1.30)	<0.001
**Waist-to-height ratio**							
<0.47	5,286	239	1.10 (0.93–1.30)		63	1.09 (0.79–1.50)	
0.48–0.49	5,219	334	1.00 (Reference)		94	1.00 (Reference)	
0.50–0.51	5,360	501	1.11 (0.96–1.27)		173	1.34 (1.04–1.72)	
0.52–0.54	5,264	663	1.07 (0.94–1.23)		238	1.31 (1.03–1.67)	
≥0.55	5,228	1,068	1.24 (1.09–1.40)	0.005	427	1.65 (1.32–2.08)	<0.001
per 0.1 unit	26,357	2,805	1.24 (1.15–1.33)	<0.001	995	1.50 (1.33–1.68)	<0.001
per SD (0.05)	26,357	2,805	1.12 (1.08–1.16)	<0.001	995	1.24 (1.16–1.31)	<0.001

Abbreviations: HR = hazard ratio, CI = confidence interval, SD = standard deviation.

a Adjusted for age (in the time scale), smoking (never, former, current, unknown) and physical activity per week (no, <3 hours light, ≥3 hours light or <1 hour hard, ≥1 hour hard, unknown).

b This category was excluded from the remainder of the analysis presented in the table.

**Table 2:** Risk of death from all causes and from cardiovascular disease among women aged 20–79; associations with anthropometric measures.

		All causes			Cardiovascular disease		
Anthropometric measures	No. of persons	No. of deaths	Adjusted^a^ HR (95% CI)	*P*_trend_	No. of deaths	Adjusted^a^ HR (95% CI)	*P*_trend_
**Body mass index (kg/m^2^)**							
<18.5^b^	314	44	2.02 (1.49–2.74)		9	1.39 (0.71–2.71)	
18.5–24.9	13,895	819	1.00 (Reference)		230	1.00 (Reference)	
25.0–29.9	10,947	872	0.81 (0.74–0.90)		308	0.93 (0.78–1.10)	
30.0–34.9	3,961	469	0.93 (0.83–1.05)		181	1.10 (0.90–1.35)	
≥35.0	1,393	204	1.24 (1.06–1.45)	0.26	74	1.41 (1.08–1.85)	0.02
per 5 kg/m^2^	30,196	2,364	1.03 (0.98–1.07)	0.27	793	1.10 (1.03–1.19)	0.009
per SD (4.5 kg/m^2^)	30,196	2,364	1.02 (0.98–1.07)	0.27	793	1.09 (1.02–1.17)	0.009
**Waist circumference (cm)**							
<70	3,981	126	1.11 (0.92–1.35)		25	0.94 (0.62–1.44)	
70–79	11,122	566	1.00 (Reference)		152	1.00 (Reference)	
80–89	8,589	761	1.00 (0.90–1.12)		265	1.14 (0.93–1.40)	
90–99	4,330	537	1.11 (0.99–1.23)		207	1.36 (1.10–1.68)	
≥100	2,174	374	1.48 (1.30–1.70)	<0.001	144	1.80 (1.43–2.27)	<0.001
per 10 cm	30,196	2,364	1.11 (1.07–1.16)	<0.001	793	1.20 (1.12–1.27)	<0.001
per SD (11.3 cm)	30,196	2,364	1.13 (1.09–1.18)	<0.001	793	1.22 (1.14–1.31)	<0.001
**Hip circumference (cm)**							
<95	6,457	348	1.10 (0.95–1.27)		96	1.17 (0.89–1.54)	
95–99	6,639	428	1.00 (Reference)		115	1.00 (Reference)	
100–104	6,840	499	0.86 (0.75–0.98)		173	1.04 (0.82–1.31)	
105–109	4,498	410	0.87 (0.76–1.00)		151	1.07 (0.84–1.36)	
≥110	5,762	679	1.02 (0.90–1.15)	0.33	258	1.27 (1.01–1.58)	0.14
per 10 cm	30,196	2,364	1.03 (0.98–1.07)	0.20	793	1.10 (1.02–1.18)	0.01
per SD (9.4 cm)	30,196	2,364	1.03 (0.99–1.07)	0.20	793	1.09 (1.02–1.17)	0.01
**Waist-to-hip ratio**							
<0.74	6,040	191	1.01 (0.84–1.22)		46	0.94 (0.65–1.35)	
0.74–0.77	6,011	282	1.00 (Reference)		83	1.00 (Reference)	
0.78–0.79	5,988	413	1.08 (0.93–1.26)		134	1.11 (0.84–1.46)	
0.80–0.83	6,125	572	1.16 (1.00–1.34)		189	1.17 (0.90–1.51)	
≥0.84	6,032	906	1.48 (1.29–1.69)	<0.001	341	1.65 (1.30–2.10)	<0.001
per 0.1 unit	30,196	2,364	1.34 (1.25–1.43)	<0.001	793	1.49 (1.33–1.66)	<0.001
per SD (0.06)	30,196	2,364	1.19 (1.15–1.24)	<0.001	793	1.27 (1.18–1.36)	<0.001
**Waist-to-height ratio**							
<0.43	6,001	156	1.29 (1.05–1.59)		30	1.29 (0.83–2.02)	
0.43–0.46	6,114	235	1.00 (Reference)		55	1.00 (Reference)	
0.47–0.49	6,010	407	1.19 (1.01–1.40)		121	1.35 (0.98–1.86)	
0.50–0.54	6,014	606	1.15 (0.99–1.34)		218	1.42 (1.05–1.91)	
≥0.55	6,057	960	1.35 (1.16–1.56)	0.005	369	1.71 (1.28–2.28)	<0.001
per 0.1 unit	30,196	2,364	1.20 (1.14–1.27)	<0.001	793	1.34 (1.21–1.47)	<0.001
per SD (0.07)	30,196	2,364	1.14 (1.10–1.19)	<0.001	793	1.23 (1.15–1.32)	<0.001

Abbreviations: HR = hazard ratio, SD = standard deviation, CI = confidence interval.

a Adjusted for age (in the time scale), smoking (never, former, current, unknown) and physical activity per week (no, <3 hours light, ≥3 hours light or <1 hour hard, ≥1 hour hard, unknown).

b This category was excluded from the remainder of the analysis presented in the table.

All cause mortality was for both sexes statistically significantly lower in the BMI range 25.0–29.9 compared to the reference group (BMI 18.5–24.9), given the above adjustments.

Overall the results were similar for both sexes except for WHR appearing as a somewhat stronger predictor among women, as compared to men, while HRs for WHtR seemed more comparable with that of waist circumference than WHR (Table 2). This was apparent in both mortality categories.

The sensitivity analyses did not deviate considerably from the primary results. Among men, the HRs per one SD increase in anthropometric measures never differed more than 0.02 from the main model (Table S2). Among women, adjustment for self-reported diabetes and alcohol consumption resulted in a slight decrease in all HRs (Table S3). The decrease was in the range of 0.04–0.06 for CVD mortality and 0.03–0.04 for total mortality. Less deviation from the main model was seen in other parts of the analysis.

Table 3 shows the χ^2^ likelihood ratio statistic for each measure. Within brackets the informativeness is given in percentage relative to WHR which was the most informative measure. The table shows results from analysis of the anthropometric measures as continuous variables. Our sensitivity analysis, examining the measures as categorical variables, gave similar results (data not shown).

**Table 3:** Relative “informativeness” of different anthropometric measures in relation to mortality; χ^2^ likelihood ratio statistics for each measure and, within brackets, percentage of χ^2^ for waist-to-hip ratio.

	Informativeness	
Anthropometric measures	All cause mortality	Cardiovascular disease mortality
**Men**		
Body mass index	1.5 (3%)	13.3 (31%)
Waist circumference	26.3 (48%)	30.4 (70%)
Hip circumference	0.1 (0.2%)	3.7 (8%)
Waist-to-hip ratio	54.7 (100%)	43.5 (100%)
Waist-to-height ratio	34.4 (63%)	45.0 (104%)
**Women**		
Body mass index	1.2 (2%)	6.6 (15%)
Waist circumference	33.4 (47%)	30.7 (69%)
Hip circumference	1.6 (2%)	6.3 (14%)
Waist-to-hip ratio	71.5 (100%)	44.4 (100%)
Waist-to-height ratio	38.7 (54%)	33.2 (75%)

The results from our analysis of reclassification and discrimination improvement are shown in Table 4 and Table 5. Among men (Table 4), WHtR offered most improvement to the prediction models studied, judging from the IDI, followed closely by WHR. Among women (Table 5), most improvement was associated with WHR, followed by WHtR and waist circumference. BMI and hip circumference seemed to add little or no information to the prediction models. Waist circumference, WHR and WHtR alternately produced the highest NRI, depending on the model and cut-points used. Some discordance between NRI and IDI estimates were noted.

**Table 4:** Risk reclassification improvement among men^a^; anthropometric measures and risk of death from cardiovascular disease.

Anthropometric measures	IDI (‰)	*P*	NRI 1^b^ (‰)	*P*	NRI 2^c^ (‰)	*P*	NRI 3^d^ (‰)	*P*
**Model A** e								
Body mass index	0.59	0.20	1.50	0.64	1.64	0.78	5.74	0.39
Waist circumference	1.99	0.009	4.32	0.28	1.09	0.88	9.62	0.23
Hip circumference	0.10	0.58	1.24	0.48	−0.79	0.81	0.73	0.84
Waist-to-hip ratio	3.45	<0.001	4.20	0.35	5.88	0.42	15.44	0.07
Waist-to-height ratio	3.64	<0.001	2.86	0.52	5.39	0.47	13.37	0.12
**Model B** f								
Body mass index	0.40	0.42	−1.94	0.61	−4.16	0.39	−2.41	0.69
Waist circumference	1.59	0.04	7.33	0.14	0.67	0.91	12.76	0.10
Hip circumference	0.09	0.72	−0.04	0.99	4.20	0.27	5.78	0.21
Waist-to-hip ratio	2.63	0.007	3.69	0.46	4.32	0.53	13.64	0.11
Waist-to-height ratio	2.77	0.005	7.23	0.17	−6.18	0.36	6.84	0.43

Abbreviations: IDI = integrated discrimination improvement, NRI = net reclassification improvement.

a Participants with body mass index <18.5 kg/m^2^ were excluded from the analysis.

b NRI when adding a given anthropometric measure to a prediction model using two risk categories (<5%, ≥5%).

c Three risk categories (<1%, 1–9%, ≥10%).

d Four risk categories (<1%, 1–4%, 5–9%, ≥10%).

e Variable included in model: Age.

f Variables included in model: Age, smoking status, systolic blood pressure, and total cholesterol.

**Table 5:** Risk reclassification improvement among women^a^; anthropometric measures and risk of death from cardiovascular disease.

Anthropometric measures	IDI (‰)	*P*	NRI 1^b^ (‰)	*P*	NRI 2^c^ (‰)	*P*	NRI 3^d^ (‰)	*P*
**Model A** e								
Body mass index	0.94	0.07	0.28	0.95	−8.41	0.23	−6.63	0.43
Waist circumference	4.12	<0.001	2.73	0.67	8.42	0.37	15.00	0.19
Hip circumference	1.12	0.03	−0.98	0.81	−7.35	0.28	−7.38	0.35
Waist-to-hip ratio	5.01	<0.001	2.15	0.77	26.76	0.009	32.21	0.01
Waist-to-height ratio	4.36	<0.001	−4.39	0.51	10.77	0.26	9.10	0.43
**Model B** f								
Body mass index	0.84	0.15	−3.27	0.42	5.90	0.35	5.36	0.48
Waist circumference	3.46	0.002	7.09	0.26	30.25	0.001	43.01	<0.001
Hip circumference	1.11	0.07	−2.80	0.53	6.36	0.34	6.98	0.38
Waist-to-hip ratio	3.90	0.002	−3.95	0.49	33.30	<0.001	36.08	<0.001
Waist-to-height ratio	3.65	0.001	4.37	0.47	25.41	0.006	35.50	0.001

Abbreviations: IDI = integrated discrimination improvement, NRI = net reclassification improvement.

a Participants with body mass index <18.5 kg/m^2^ were excluded from the analysis.

b NRI when adding a given anthropometric measure to a prediction model using two risk categories (<5%, ≥5%).

c Three risk categories (<1%, 1–9%, ≥10%).

d Four risk categories (<1%, 1–4%, 5–9%, ≥10%).

e Variable included in model: Age.

f Variables included in model: Age, smoking status, systolic blood pressure, and total cholesterol.

Risk of death from CVD associated with the measures studied stratified by age is shown in Table 6. HRs are given both for men and women, aged 20–59 years versus 60–79 at baseline, for each of the measures. The strongest association was between CVD mortality and WHtR for men and WHR for women. For all measures the HRs were somewhat higher for the younger stratum.

**Table 6:** Risk of death from cardiovascular disease among men and women aged 20–79 years^a^; associations with anthropometric measures stratified by age at baseline.

	Adjusted^b^ HR (95% CI)			
	Men		Women	
Anthropometric measures	20–59 years	60–79 years	20–59 years	60–79 years
Body mass index, per 5 kg/m^2^	1.55 (1.27–1.89)	1.11 (1.00–1.23)	1.26 (0.97–1.64)	1.09 (1.01–1.18)
Waist circumference, per 10 cm	1.49 (1.28–1.74)	1.15 (1.07–1.24)	1.36 (1.12–1.66)	1.18 (1.10–1.26)
Hip circumference, per 10 cm	1.45 (1.14–1.84)	1.04 (0.93–1.17)	1.12 (0.86–1.47)	1.09 (1.01–1.18)
Waist-to-hip ratio, per 0.1 unit	1.96 (1.52–2.53)	1.35 (1.20–1.51)	2.15 (1.60–2.89)	1.42 (1.26–1.60)
Waist-to-height ratio, per 0.1 unit	2.25 (1.73–2.93)	1.37 (1.21–1.55)	1.69 (1.23–2.33)	1.30 (1.18–1.44)
**Per one SD increase**				
Body mass index^c^	1.35 (1.18–1.55)	1.08 (1.00–1.15)	1.23 (0.97–1.56)	1.08 (1.01–1.16)
Waist circumference^d^	1.44 (1.26–1.66)	1.14 (1.06–1.22)	1.42 (1.14–1.77)	1.20 (1.12–1.30)
Hip circumference^e^	1.25 (1.08–1.45)	1.03 (0.96–1.10)	1.11 (0.86–1.44)	1.09 (1.01–1.17)
Waist-to-hip ratio^f^	1.47 (1.27–1.69)	1.18 (1.11–1.26)	1.58 (1.33–1.89)	1.24 (1.15–1.33)
Waist-to-height ratio^g^	1.54 (1.34–1.76)	1.18 (1.10–1.26)	1.46 (1.16–1.84)	1.21 (1.13–1.30)

Abbreviations: HR = hazard ratio, SD = standard deviation, CI = confidence interval.

a Participants with body mass index <18.5 kg/m^2^ were excluded from the analyses.

b Adjusted for age (in the time scale), smoking (never, former, current, unknown) and physical activity per week (no, <3 hours light, ≥3 hours light or <1 hour hard, ≥1 hour hard, unknown).

c One SD: men 3.5 kg/m^2^, women 4.5 kg/m^2^.

d One SD: men 9.2 cm, women 11.5 cm.

e One SD: men 9.2 cm, women 9.4 cm.

f One SD: 0.06 for both sexes.

g One SD: men 0.05, women 0.07.

The results for CVD mortality from the mutual adjustment analysis of hip and waist circumferences are shown in Table 7. Adjusting for hip circumference strengthened the association of waist girth with CVD mortality considerably for both sexes. Increasing hip circumference, on the other hand, seemed to be protective when adjusting for the waist. The results were similar for all cause mortality (not shown in table). The results from a corresponding analysis of BMI and WHR, mutually adjusted, are shown in Table 8. Adjusting BMI for WHR attenuated the association of BMI with mortality significantly, while adjusting WHR for BMI had no significant effect on the association.

**Table 7:** Risk of death from cardiovascular disease among men and women aged 20–79 years^a^; associations with waist and hip circumferences mutually adjusted.

		Men			Women		
Anthropometric measures		No. of persons	No. of deaths	Adjusted^b^ HR (95% CI)	No. of persons	No. of deaths	Adjusted^b^ HR (95% CI)
**Waist circumference (cm)**							
**Men**	**Women**						
<80	<70	1,882	33	0.98 (0.67–1.42)	3,981	25	0.86 (0.56–1.32)
80–89	70–79	9,466	233	1.00 (Reference)	11,122	152	1.00 (Reference)
90–99	80–89	10,378	404	1.20 (1.00–1.44)	8,589	265	1.26 (1.02–1.56)
100–109	90–99	3,625	226	1.52 (1.20–1.93)	4,330	207	1.65 (1.28–2.14)
≥110	≥100	1,006	99	2.64 (1.91–3.67)	2,174	144	2.54 (1.81–3.58)
Waist circumference, per 10 cm		26,357	995	1.42 (1.28–1.58)	30,196	793	1.44 (1.29–1.61)
**Hip circumference (cm)**							
<95		2,360	80	1.16 (0.89–1.50)	6,457	96	1.40 (1.06–1.84)
95–99		6,158	225	1.00 (Reference)	6,639	115	1.00 (Reference)
100–104		9,203	335	0.75 (0.63–0.90)	6,840	173	0.88 (0.69–1.12)
105–109		5,471	200	0.61 (0.49–0.76)	4,498	151	0.77 (0.60–1.01)
≥110		3,165	155	0.65 (0.49–0.86)	5,762	258	0.67 (0.50–0.91)
Hip circumference, per 10 cm		26,357	995	0.73 (0.62–0.86)	30,196	793	0.77 (0.68–0.88)

Abbreviations: HR = hazard ratio, CI = confidence interval.

a Participants with body mass index <18.5 kg/m^2^ were excluded from all analyses.

b Adjusted for age (in the time scale), smoking (never, former, current, unknown), physical activity per week (no, <3 hours light, ≥3 hours light or <1 hour hard, ≥1 hour hard, unknown), and either waist circumference or hip circumference.

**Table 8:** Risk of death from cardiovascular disease among men and women aged 20–79 years^a^; associations with body mass index and waist-to-hip ratio mutually adjusted.

		Men			Women		
Anthropometric measures		No. of persons	No. of deaths	Adjusted^b^ HR (95% CI)	No. of persons	No. of deaths	Adjusted^b^ HR (95% CI)
**Body mass index (kg/m^2^)**							
18.5–24.9		9,575	300	1.00 (Reference)	13,895	230	1.00 (Reference)
25.0–29.9		13,138	492	0.91 (0.78–1.07)	10,947	308	0.81 (0.68–0.97)
30.0–34.9		3,154	175	1.08 (0.87–1.34)	3,961	181	0.88 (0.71–1.08)
≥35.0		490	28	1.23 (0.81–1.86)	1,393	74	1.07 (0.81–1.42)
per SD (M: 3.4, W: 4.5)		26,357	995	1.01 (0.94–1.09)	30,196	793	1.00 (0.93–1.08)
**Waist-to-hip ratio**							
**Men**	**Women**						
<0.85	<0.74	5,286	75	1.14 (0.85–1.53)	6,040	46	0.82 (0.55–1.21)
0.86–0.87	0.74–0.77	5,219	97	1.00 (Reference)	6,011	83	1.00 (Reference)
0.88–0.89	0.78–0.79	5,360	167	1.25 (0.96–1.62)	5,988	134	1.15 (0.87–1.51)
0.90–0.93	0.80–0.83	5,264	233	1.35 (1.07–1.70)	6,125	189	1.11 (0.88–1.40)
≥0.94	≥0.84	5,228	423	1.64 (1.30–2.07)	6,032	341	1.59 (1.27–2.00)
per SD (both sexes: 0.06)		26,357	995	1.22 (1.14–1.31)	30,196	793	1.27 (1.18–1.36)

Abbreviations: HR = hazard ratio, CI = confidence interval, SD = standard deviation.

a Persons with body mass index <18.5 kg/m^2^ were excluded from all analyses.

b Adjusted for age (in the time scale), smoking (never, former, current, unknown), physical activity per week (no, <3 hours light, ≥3 hours light or <1 hour hard, ≥1 hour hard, unknown), and either body mass index or waist-to-hip ratio.

## Discussion

Of the five anthropometric measures studied, WHR and WHtR were most strongly associated with mortality, after adjusting for confounding factors. This was true both regarding overall mortality and death from CVD specifically. In accordance with other studies, our results show that BMI is a poorer predictor of death than the other measures [2]–[7], [10]–[13], [40]. These results underscore the advantage of assessing body configuration rather than body weight when estimating mortality risk. Furthermore, when controlling for waist circumference, increasing hip circumference appears to be protective in both genders. In our study, obesity emerged as a more important risk factor among young people, in comparison to older. This is in coherence with earlier studies [41].

In all parts of our analysis, BMI showed weaker associations with both all cause mortality and CVD mortality, when compared to waist circumference, WHR and WHtR. Furthermore, BMI was the only among these four measures which failed to show a statistically significant association with all cause mortality. BMI also contributed less additional information to the prediction models studied (Tables 4 and 5), and offered poorer fitting models (Table 3). Hence, BMI seems to be a poorer indicator of disease risk than the other measures studied, being superior only to hip circumference. When adjusting for WHR, BMI seemed even less predictive, while adjusting for BMI had no effect on WHR mortality associations. This emphasises the superiority of the alternative measures over BMI as indicators of CVD risk.

Waist circumference proved to be a statistically significant risk factor in all analyses, but still showed weaker associations with mortality than both WHR and WHtR. In particular, it emerged as a strong risk factor when adjusting for hip circumference. This underlines the significance of considering body configuration rather than the abdominal girth alone.

Hip circumference showed a weak positive association with mortality. However, when adjusting for waist circumference, it proved to be inversely related to CVD mortality in both genders. This finding is in accordance with previous research [9], [13], [16]–[18].

Both in the presence (Table 6) and absence of age stratification (Tables 1 and 2), WHR turned out to be a stronger risk factor than WHtR among women, whilst the two measures had similar predictive power among men. This gender difference favoured the use of WHtR among younger men. In any case, our results show that WHR and WHtR are superior to the other measures in relation to prediction of mortality.

Based on the IDIs (Tables 4 and 5), WHR and WHtR offered the greatest improvement to our prediction models, followed closely by waist circumference for women. The improvement was in the range of 2–5‰. In comparison, smoking and systolic blood pressure produced IDIs in the range of 5–6‰ for men and 1.7–2.5‰ for women, using the same models. Some discrepancies was noted between the IDIs and the NRIs (e.g. a negative NRI 2 for WHtR among men, Table 4). This can be explained by the choice of cut-points in combination with low precision of the NRI estimates. The variation in NRIs highlights the importance of careful selection of cut-points, depending on the purpose. Identification of optimal cut-points depends on chosen background factors as well as considerations related to clinical relevance. Our results indicate that the best discrimination is obtained by use of waist circumference, WHR or WHtR.

The main strength of our investigation lies in the prospective and comprehensive nature of the HUNT 2 study, its good participation rates, and it being fairly representative for the entire Norwegian nation. The fact that the HUNT population is ethnically homogenous may also be considered a strength in this context, since ethnic differences (genetic and epigenetic factors) may influence the predictive properties of anthropometric measures [42]–[44].

The HUNT 2 database lacks comprehensive information on the participants' dietary habits and cancer history. However, the exclusion of participants with BMI <18.5 kg/m^2^ and the sensitivity analysis which excludes the first three years of follow-up minimise the potential for confounding by cancer. Our sensitivity analysis indicates that the impact of other potential confounders is minimal.

Our study adds further knowledge to the evidence that BMI is not the most appropriate measure of obesity in everyday clinical practice. WHR is as easy to calculate as BMI and is presently better documented than WHtR. It therefore appears reasonable to recommend WHR as the primary measure of body composition and obesity, at least when it comes to assessing risk of CVD. There is, however, need for further clarification before determining whether WHtR should be considered an even better alternative than WHR. Single (waist circumference in isolation) or additional measures (involving weight and/or height) may also be added to nuance estimations of CVD risk when indicated, for instance in relation to clearly obese or under-weight individuals with a favourable WHR. A certain weakness of the approach suggested here is the documented, inter-personal variance in measurement of waist and hip circumferences [45]. This problem can be addressed by standardised measurement procedures [46] and adequate training [45].

It is hard to determine how much effort should be put into training healthcare workers to measure WHR or WHtR in a standardised and reproducible manner, as the potential for predictive improvement will depend on the selected cut-off points and also the choice of prediction model. In relation to combined risk algorithms [47], [48], our results indicate that a NRI of up to 4% might be reached for women and 1.5% for men, depending on cut-off points, by replacing BMI with waist circumference, WHR or WHtR. Identification of the most appropriate cut-offs for a given prediction model could eventually be addressed in a future study. Most preventive CVD guidelines [33], [34], [49], [50] however do not include markers of obesity in their combined risk algorithms. Authoritative guidelines currently treat body composition/configuration as an isolated risk factor and usually lack clear specifications regarding the numerical impact on disease risk. As long as this approach is recommended for use in clinical practice, we argue for the use of the anthropometric measure with the best predictive properties. From this perspective, it appears rational to replace BMI by WHR or WHtR when evaluating CVD mortality risk.

## Supporting Information

Table S1 Baseline characteristics of the study population.
(DOCX)

Table S2 Risk of death from all causes and from cardiovascular disease among men aged 20–79; associations with anthropometric measures (hazard ratios per increase in anthropometric measures of one standard deviation). Sensitivity analysis involving different models.
(DOCX)

Table S3 Risk of death from all causes and from cardiovascular disease among women aged 20–79; associations with anthropometric measures (hazard ratios per increase in anthropometric measures of one standard deviation). Sensitivity analysis involving different models.
(DOCX)
