# Supplementary material for: Body Configuration as a Predictor of Mortality: Comparison of Five Anthropometric Measures in a 12 Year Follow-Up of the Norwegian HUNT 2 Study
Source: PLoS One. 2011 Oct 20;6(10):e26621. doi: 10.1371/journal.pone.0026621 (PMC3197688; doi:10.1371/journal.pone.0026621)
Supplement: Table S1 — Baseline characteristics of the study population. (DOCX) [file pone.0026621.s001.docx]

**Supporting information**

**Table S1.** Baseline characteristics of the study population aged 20-79^a^.

| **Variable** | **Men (N = 26,463)** | | **Women (N = 30,505)** | |
| --- | --- | --- | --- | --- |
| Mean age, years (SD) | 46.7 | (15.1) | 47.5 | (15.6) |
| Mean weight, kg (SD) | 83.6 | (12.2) | 70.8 | (12.4) |
| Mean body mass index, kg/m^2^ (SD) | 26.4 | (3.4) | 26.2 | (4.5) |
| Mean waist circumference, cm (SD) | 91.5 | (9.1) | 81.1 | (11.3) |
| Mean hip circumference, cm (SD) | 102.3 | (6.2) | 101.9 | (9.4) |
| Mean waist-to-hip ratio (SD) | 0.89 | (0.06) | 0.79 | (0.06) |
| Mean waist-to-height ratio (SD) | 0.51 | (0.05) | 0.49 | (0.07) |
| Mean systolic blood pressure, mmHg (SD) | 138.9 | (18.0) | 133.8 | (22.3) |
| Mean serum cholesterol, mmol/L (SD) | 5.8 | (1.2) | 5.9 | (1.3) |
| Diabetes mellitus^b^ (%) | 564 | (2.1) | 610 | (2.0) |
| University degree (%) | 5,392 | (21.1) | 6,153 | (20.3) |
| Smoking (%) |  |  |  |  |
| Never smokers | 10,298 | (39.1) | 13,595 | (45.0) |
| Current smokers | 7,582 | (28.8) | 5,680 | (18.8) |
| Former smokers | 7,224 | (27.4) | 9,116 | (30.2) |
| Unknown smoking status | 1,253 | (4.8) | 1,805 | (6.0) |
| Physical activity^c^ (%) |  |  |  |  |
| No activity | 1,810 | (6.9) | 1,712 | (5.7) |
| <3 h easy | 6,157 | (23.4) | 9,664 | (32.0) |
| 3+ h easy, <1 h hard | 8,100 | (30.7) | 9,409 | (31.2) |
| 1+ h hard | 8,556 | (32.5) | 6,275 | (20.8) |
| Unknown | 1,734 | (6.6) | 3,136 | (10.4) |
| Alcohol consumption, glasses/week^d^ (%) |  |  |  |  |
| Total abstinence | 1,756 | (6.7) | 4,050 | (13.4) |
| 0-2 | 14,983 | (56.8) | 21,675 | (71.8) |
| 2.1-5 | 6,718 | (25.5) | 3,801 | (12.6) |
| 5.1-8 | 2,384 | (9.0) | 619 | (2.0) |
| >8 | 516 | (2.0) | 51 | (0.2) |

SD = standard deviation.

^a^Participants with body mass index lower than 18.5 kg/m^2^ are not included in the table.

^b^Number of persons with self-reported diabetes.

^c^Self-reported physical activity per week.

^d^Self-reported weekly alcohol consumption (beer, wine, strong liqour), number of glasses.
