# Supplementary material for: Body Configuration as a Predictor of Mortality: Comparison of Five Anthropometric Measures in a 12 Year Follow-Up of the Norwegian HUNT 2 Study
Source: PLoS One. 2011 Oct 20;6(10):e26621. doi: 10.1371/journal.pone.0026621 (PMC3197688; doi:10.1371/journal.pone.0026621)
Supplement: Table S2 — Risk of death from all causes and from cardiovascular disease among men aged 20–79; associations with anthropometric measures (hazard ratios per increase in anthropometric measures of one standard deviation). Sensitivity analysis involving different models. (DOCX) [file pone.0026621.s002.docx]

**Table S2.** Risk of death from all causes and from cardiovascular disease among men aged 20-79^a^; associations with anthropometric measures (hazard ratios per increase in anthropometric measures of one standard deviation^b^). Sensitivity analysis involving different models.

|  |  | **All causes** | | | **Cardiovascular disease** | | |
| --- | --- | --- | --- | --- | --- | --- | --- |
| **Anthropometric measures** | **No. of persons** | **No. of deaths** | **Adjusted HR (95% CI)** | ***P*_trend_** | **No. of deaths** | **Adjusted HR (95% CI)** | ***P*_trend_** |
| **Model 2^c^** | 25,104 | 2,621 |  |  | 919 |  |  |
| Body mass index |  |  | 1.03 (0.99-1.07) | 0.11 |  | 1.13 (1.06-1.21) | <0.001 |
| Waist circumference |  |  | 1.10 (1.06-1.15) | <0.001 |  | 1.20 (1.13-1.28) | <0.001 |
| Hip circumference |  |  | 1.01 (0.97-1.05) | 0.73 |  | 1.06 (0.99-1.13) | 0.08 |
| Waist-to-hip ratio |  |  | 1.15 (1.11-1.19) | <0.001 |  | 1.25 (1.17-1.33) | <0.001 |
| Waist-to-height ratio |  |  | 1.13 (1.08-1.17) | <0.001 |  | 1.25 (1.18-1.33) | <0.001 |
| **Model 3^d^** | 26,357 | 2,805 |  |  | 995 |  |  |
| Body mass index |  |  | 1.02 (0.98-1.06) | 0.38 |  | 1.12 (1.05-1.19) | 0.001 |
| Waist circumference |  |  | 1.09 (1.05-1.13) | <0.001 |  | 1.18 (1.11-1.25) | <0.001 |
| Hip circumference |  |  | 1.00 (0.96-1.04) | 0.87 |  | 1.05 (0.99-1.12) | 0.11 |
| Waist-to-hip ratio |  |  | 1.14 (1.10-1.18) | <0.001 |  | 1.22 (1.15-1.29) | <0.001 |
| Waist-to-height ratio |  |  | 1.11 (1.07-1.15) | <0.001 |  | 1.22 (1.15-1.30) | <0.001 |
| **Model 4^e^** | 25,942 | 2,390 |  |  | 835 |  |  |
| Body mass index |  |  | 1.04 (1.00-1.09) | 0.05 |  | 1.12 (1.05-1.20) | <0.001 |
| Waist circumference |  |  | 1.11 (1.07-1.16) | <0.001 |  | 1.19 (1.11-1.27) | <0.001 |
| Hip circumference |  |  | 1.02 (0.98-1.06) | 0.41 |  | 1.08 (1.00-1.15) | 0.04 |
| Waist-to-hip ratio |  |  | 1.15 (1.11-1.20) | <0.001 |  | 1.22 (1.14-1.30) | <0.001 |
| Waist-to-height ratio |  |  | 1.13 (1.09-1.18) | <0.001 |  | 1.23 (1.15-1.32) | <0.001 |

Abbreviations: HR = hazard ratio, CI = confidence interval.

^a^Participants with body mass index lower than 18.5 kg/m^2^ were excluded from all analyses.

^b^Body mass index: 3.4 kg/m^2^; Waist circumference: 9.1 cm; Hip circumference 6.2 cm; Waist-to-hip ratio: 0.06; Waist-to-height ratio: 0.05.

^c^Model 2: Adjusted for age (in the time scale), smoking (never, former, current), and physical activity per week (no, <3 hours light, ≥3 hours light or <1 hour hard, ≥1 hour hard, unknown). Participants with unknown smoking status were excluded.

^d^Model 3: Adjusted for age, smoking (never, former, current, unknown), physical activity, diabetes mellitus (yes, no), and weekly alcohol consumption (abstinence, 0-2 glasses [units], 2.1-5 glasses, 5.1-8 glasses, >8).

^e^Model 4: Adjusted for age, smoking, (never, former, current, unknown), and physical activity. The first three years of follow up were excluded.
