# Supplementary material for: Body Configuration as a Predictor of Mortality: Comparison of Five Anthropometric Measures in a 12 Year Follow-Up of the Norwegian HUNT 2 Study
Source: PLoS One. 2011 Oct 20;6(10):e26621. doi: 10.1371/journal.pone.0026621 (PMC3197688; doi:10.1371/journal.pone.0026621)
Supplement: Table S3 — Risk of death from all causes and from cardiovascular disease among women aged 20–79; associations with anthropometric measures (hazard ratios per increase in anthropometric measures of one standard deviation). Sensitivity analysis involving different models. (DOCX) [file pone.0026621.s003.docx]

**Table S3.** Risk of death from all causes and from cardiovascular disease among women aged 20-79^a^; associations with anthropometric measures (hazard ratios per increase in anthropometric measures of one standard deviation^b^). Sensitivity analysis involving different models.

|  |  | **All causes** | | | **Cardiovascular disease** | | |
| --- | --- | --- | --- | --- | --- | --- | --- |
| **Anthropometric measures** | **No. of persons** | **No. of deaths** | **Adjusted HR (95% CI)** | ***P*_trend_** | **No. of deaths** | **Adjusted HR (95% CI)** | ***P*_trend_** |
| **Model 2^c^** | 28,391 | 2,104 |  |  | 696 |  |  |
| Body mass index |  |  | 1.02 (0.98-1.07) | 0.30 |  | 1.12 (1.05-1.21) | 0.002 |
| Waist circumference |  |  | 1.13 (1.08-1.18) | <0.001 |  | 1.24 (1.15-1.34) | <0.001 |
| Hip circumference |  |  | 1.02 (0.98-1.07) | 0.28 |  | 1.12 (1.04-1.21) | 0.01 |
| Waist-to-hip ratio |  |  | 1.19 (1.14-1.24) | <0.001 |  | 1.26 (1.17-1.36) | <0.001 |
| Waist-to-height ratio |  |  | 1.14 (1.09-1.19) | <0.001 |  | 1.26 (1.17-1.36) | <0.001 |
| **Model 3^d^** | 30,196 | 2,364 |  |  | 793 |  |  |
| Body mass index |  |  | 0.99 (0.95-1.03) | 0.69 |  | 1.05 (0.98-1.12) | 0.19 |
| Waist circumference |  |  | 1.09 (1.05-1.14) | <0.001 |  | 1.16 (1.08-1.25) | <0.001 |
| Hip circumference |  |  | 1.00 (0.96-1.04) | 0.87 |  | 1.05 (0.97-1.12) | 0.21 |
| Waist-to-hip ratio |  |  | 1.16 (1.12-1.21) | <0.001 |  | 1.23 (1.15-1.31) | <0.001 |
| Waist-to-height ratio |  |  | 1.10 (1.06-1.15) | <0.001 |  | 1.17 (1.09-1.26) | <0.001 |
| **Model 4^e^** | 29,907 | 2,075 |  |  | 699 |  |  |
| Body mass index |  |  | 1.02 (0.98-1.07) | 0.27 |  | 1.08 (1.01-1.17) | 0.04 |
| Waist circumference |  |  | 1.13 (1.08-1.18) | <0.001 |  | 1.22 (1.13-1.32) | <0.001 |
| Hip circumference |  |  | 1.02 (0.98-1.07) | 0.28 |  | 1.08 (1.00-1.16) | 0.04 |
| Waist-to-hip ratio |  |  | 1.19 (1.14-1.24) | <0.001 |  | 1.28 (1.19-1.38) | <0.001 |
| Waist-to-height ratio |  |  | 1.14 (1.09-1.19) | <0.001 |  | 1.23 (1.15-1.33) | <0.001 |

Abbreviations: HR = hazard ratio, CI = confidence interval.

^a^Participants with body mass index lower than 18.5 kg/m^2^ were excluded from all analyses.

^b^Body mass index: 4.5 kg/m^2^; Waist circumference: 11.3 cm; Hip circumference 9.4 cm; Waist-to-hip ratio: 0.06; Waist-to-height ratio: 0.07.

^c^Model 2: Adjusted for age (in the time scale), smoking (never, former, current), and physical activity per week (no, <3 hours light, ≥3 hours light or <1 hour hard, ≥1 hour hard, unknown). Participants with unknown smoking status were excluded.

^d^Model 3: Adjusted for age, smoking (never, former, current, unknown), physical activity, diabetes mellitus (yes, no), and weekly alcohol consumption (abstinence, 0-2 glasses [units], 2.1-5 glasses, 5.1-8 glasses, >8).

^e^Model 4: Adjusted for age, smoking, (never, former, current, unknown), and physical activity. The first three years of follow up were excluded.
